# The Impacts of Young Consumers’ Health Values on Functional Beverages Purchase Intentions

**DOI:** 10.3390/ijerph17103479

**Published:** 2020-05-16

**Authors:** Hsiao-Ping Chang, Chun-Chieh Ma, Han-Shen Chen

**Affiliations:** 1 Department of Health Diet and Industry Management, Chung Shan Medical University, No.110, Sec. 1, Jianguo N. Rd. Taichung City 40201, Taiwan; pamela22@csmu.edu.tw; 2 Department of Medical Management, Chung Shan Medical University Hospital, No. 110, Sec. 1, Jianguo N. Rd., Taichung City 40201, Taiwan; 3 Department of Public Administration and Management, National University of Tainan, Taiwan, No.33, Sec. 2, Shu-Lin St., Tainan 70005, Taiwan; ccma@mail.nutn.edu.tw

**Keywords:** eating patterns, consumer preferences, health food, self-efficacy, health orientation

## Abstract

Internationally, there is increasing recognition of the importance of proper diet values and habits, a balanced intake of healthy food products, and rates of obesity control encompassing information on fat content and calories. In this context, some beverage manufacturers have shifted to marketing their products as having fewer calories and more functional benefits. This study aims to develop an extended value–attitude–behavior (VAB) research model that includes three constructs, namely, cue to action, self-efficacy, and health orientation, to explore the impacts of university students’ health values on their purchase intentions concerning functional beverages. The results indicated that university students’ interest in functional beverages was significantly affected by their health values. Besides, both interests in functional beverages and health orientation were significant predictors of purchase intention, while cue to action and self-efficacy were not. Based on these results, enhancing consumers’ education about food security is suggested. Furthermore, the findings provide crucial insights for marketing channels, suggesting the beverage industry can target consumers’ health values concerning health beverages as the key to purchase intention and attract business by developing practical marketing strategies.

## 1. Introduction

According to some studies [1,2], with the gradual increase of chronic civilized diseases, obesity, and cardiovascular diseases all over the world, consumers have started to pay more attention to health, and the promotion of good value is gradually adding visibility to the notion of healthy diets. Without the parents’ supervision of diet and lifestyle, being overweight or obese has become prevalent among college students in Taiwan [3]. This encourages businesses to take advantage of business opportunities related to “functional food” and nutrient and dietary supplements.

Every country has different terms and definitions for health food (e.g., ”health food” in Taiwan, “food for specified health uses” in Japan, and “functional food” in China). In Taiwan, “health food” refers to food that contains a specific nutrient or one that produces particular health effects, but not offered to the public as treatment or remedy for human diseases. The term “functional beverages” mostly refers to beverages that contain additives to reduce blood lipids, reduce fat absorption, and aid digestion and other body functions.

In 2017, the Industrial Development Bureau, Ministry of Economic Affairs, approved a total of 379 health food items, of which 102 were beverages. Given their popularity, these beverages have a crucial role in marketing sales. In Taiwan, for example, functional beverages yield two billion NT dollars in market sales each year, which means they have outsold carbonated drinks and become the largest beverage market. This shows that consumers tend to choose “fewer calories, more function” products. Accordingly, beverages with “healthy food” labels have become the trend in the current packaged beverage market [4]. For these reasons, how to retain customers and attract new ones has become an essential issue for the industry.

Among the models that were proposed by social psychologists in the last few decades to predict and understand human behaviors, the theory of planned behavior (TPB) has gained greater acceptance. [5,6]. Yazdanpanah, Forouzani, and Hojjati [7] extended TPB with various variables (e.g., moral standards and self-identity) to investigate Iranian students’ intention to purchase organic food. Lorenz, Hartmann, and Simons [8] utilized TPB to explore consumers’ plan to buy products with origin labels, and Yadav and Pathak [9] applied it to study consumers’ green purchase behavior in developing nations. Wong, Hsu, and Chen [10] utilized extended TPB to explore consumers’ attitudes and purchase intentions for suboptimal food.

According to social adaptation theory, values, as an amalgam of social cognitions, enable individuals to adapt to an environment, guiding them to how to act in a particular situation [11,12]. It is subjective and will be formed through the social and psychological development of consumers. Scholars have addressed values to be the most abstract constructs that build attitudes and behaviors [13,14]. However, several studies proposed that cost is a more fundamental social cognition than mentality [15], and through the establishment of value, eating habits and behavior might be changed in the long term [16]. A growing number of research studies have used the value–attitude–behavior (VAB) model to analyze consumers’ purchase behavior. Honkanen, Verplanken, and Olsen [17] considered moral cognition in their exploration of whether consumers’ moral values about the environment and animal welfare affected their choices of organic food. Kang, Jun, and Arendt [18] applied the VAB model to investigate purchase intentions for a low-calorie diet. Jun et al. [16] used the model to study the effects of health value on healthful food selection intention. Although previous studies have addressed similar topics in other countries, we sought to explore the critical factor in college students’ consumption of functional beverages in Taiwan. This is the motivation of this study.

The study variables are a cue to action, self-efficacy, and health orientation. They were selected for the following reasons. Among various factors affecting consumer purchase intention, signal to work is a valuable information source as it might influence consumers’ health behavior [19,20,21]. Cue to action refers to the stimulus that urges individuals to take steps, which can be divided into external stimuli and internal stimuli. External stimuli such as media dissemination, interpersonal interaction, advice from family and friends, etc., and internal stimuli such as conscious physical discomfort, symptoms of the disease, etc., all affect whether an individual takes action or not [22,23]. Hanson and Benedict [24] indicated that cue to action has a positive influence on senior adults’ food-handling behaviors, promotes healthy behavior, and may also affect purchase intention for functional beverages.

Self-efficacy is a variable commonly seen in previous studies, and it appears in the health belief model in related research. Self-efficacy refers to a person’s confidence that he or she has enough power to do something [25]. Several studies have explored self-efficacy and purchase intention. For instance, Milne, Sheeran, and Orbell [26] showed that self-efficacy and purchase intention have a significant relationship, and Yazdanpanah et al. [7] found that self-efficacy has a positive influence on organic food purchase intention.

The third variable, health orientation, is a personal attitude toward health, beliefs, and behavior—it extends to individual concern about health-related issues [27]. De Marchi, Caputo, Nayga, and Banterle [28] demonstrated that levels of engagement in health-related action and food consumption decisions result from one’s health orientation. De Boer, McCarthy, Cowan, and Ryan [29] also found that a consumer’s health orientation hurts purchase intention for convenience foods.

Above all, this study utilizes the VAB model and considers the three research dimensions of the cue to action, self-efficacy, and health orientation. We aim to determine whether consumers’ good value affects their interest in functional beverages, as well as whether interest in functional beverages affects purchase intention. We hope that the findings will provide insights for marketing by revealing what consumers care about in their purchasing of functional beverages and thus inspire effective marketing strategies and create more business opportunities for related business organizations.

## 2. Materials and Methods

### 2.1. Research Framework 

**Figure 1:**
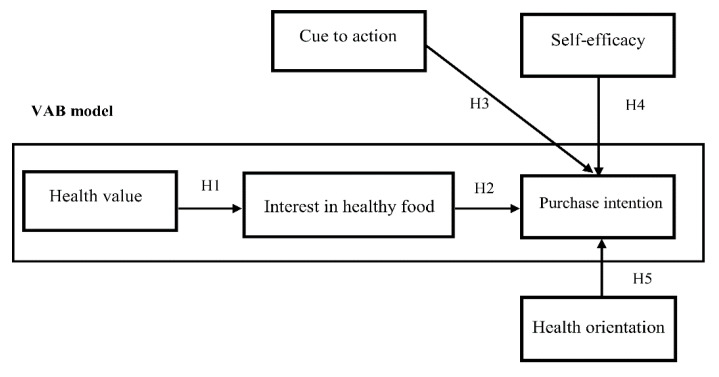
Research framework based on the value–attitude–behavior (VAB) model.

According to the VAB model, value affects behavior intention through attitude. In this study, the discussion of the relationship between consumers’ health value, interest in healthy food, and purchase intention focuses on three constructs: cue to action, self-efficacy, and health orientation. The proposed theoretical framework is shown in Figure 1, and the five hypotheses are below.

### 2.2. Research Hypotheses

The VAB model has been widely used to understand consumer behavior in the study of social psychology [15]. The underlying meaning of value is a basic standard to guide people’s actions. It is subjective and is formed through the social and psychological development of consumers. Attitude is the feeling a person has in carrying out specific behavior, or positive or negative thinking that accompanies the particular action. It is an essential factor to forecast consumer behavior intention [21], and it influences individual behavior [30]. Tudoran, Olsen, and Dopico [31] argued that value affects consumer behavior through attitude. This study utilizes health value, interest in healthy food, and purchase intention as the theoretical basis for the VAB model.

Purchase intention means the possibility of a consumer buying a product, and a higher purchase intention means a higher rate of purchasing the product [32,33]. Purchase intention is often considered an indicator of subsequent purchase and can be used to predict purchasing behavior. Lessa et al. [34] found that when consumers pay more attention to health issues, they are willing to give up high-fat or high-calorie foods for the sake of their health. Consumers’ concerns about food quality and personal hygiene have been related to them to acquire information about the quality of food, particularly at the purchase decision stage of the buying process [35]. Kang et al. [18] found that consumers choose healthy food to keep healthy and fit, and they make choices for a healthy diet using individual subjective knowledge. Food safety often linked to human health and food quality, and it plays a vital role in consumer choices is represented (e.g., expiry dates and the presence of specific ingredients and additives) [36]. Consumers’ purchase intentions become more potent when they find that eating healthy food can help them achieve their health goals (e.g., maintaining or losing weight) [37].

Tudoran et al. [31] described health value as the extent to which a person cares about his or her health status, and noted that consumers’ health value is related to product function and purchase intention. Jun, Kang, and Arendt [38] found that consumers set the goal of achieving health, so there will be different degrees of values. Moreover, health value has a positive influence on health behavior and attitude toward health products [39]. Accordingly, we hypothesize that:

**Hypothesis** **(H1).** *Health value has a positive influence on interest in functional beverages.*

Roininen, Lähteenmäki, and Tuorila [40] defined “interest in healthy food” as an interest in energy-reduced foods such as low-fat, low-calorie, and sugar-free foods. Consumers think that these can help them maintain or achieve better health, so they feel less guilty about eating a healthy diet compared to other food [41]. Tudoran, Scholderer, and Brunsø [42] indicated that the higher the emphasis consumers place on health, the more likely they are to choose healthy foods [43]. Vázquez, Curia, and Hough [44] showed that consumers who have a healthy life are more likely to be interested in continuing to eat healthy foods. The discussion results into the following hypotheses:

**Hypothesis** **(H2).** *Interest in healthy food has a positive influence on purchase intention.*

Cues to action include internal cues (e.g., disease situation) and extrinsic cues (e.g., public media report, interpersonal interaction, and health check results) that prompt the consumer to engage in health behavior [19]. A cue to action can also be a motivation behind a person’s health behavior [20]. Hanson and Benedict [24] found that cue to action has a positive effect on consumer behavior in handling food security and promoting the healthy practice. Accordingly, we hypothesize that:

**Hypothesis** **(H3).** *Cue to action has a positive influence on purchase intention.*

Bandura [18] defined self-efficacy as the confidence that one can do something. Self-efficacy has no relationship with personal skill; instead, it is related to self-judgment of the extent of one’s ability. Self-efficacy can determine individual behavior in specific situations, ways of thinking, and emotional reactions. Yazdanpanah et al. [7] found that self-efficacy positively influences purchase intention for organic food, and Milne et al. [26] showed that self-efficacy is one of the critical factors that affect purchase intention. The discussion results into the following hypotheses:

**Hypothesis** **(H4).** *Self-efficacy has a positive influence on purchase intention.*

Dutta et al. [27] defined health orientation as attitudes, beliefs, behaviors, and care about health-related issues concerning personal health. People have an incentive to engage in the healthy practice for the sake of their health [45]. Previous studies showed that health orientation influences people’s levels of food consumption related to health promotion and decision making [17,46]. The discussion results into the following hypotheses:

**Hypothesis** **(H5).** *Health orientation has a positive influence on purchase intention.*

### 2.3. Questionnaire Design

The design of questionnaire items stemmed from a review of pertinent literature and involved the use of a 7-point Likert scale, with 7 representing Strongly Agree and 1 representing Strongly Disagree. Details about the source of the questionnaire items are highlighted in Table 1. 

### 2.4. Sample Size and Composition 

The participants were students from 10 colleges (undergraduate and graduate) in central Taiwan. We chose college students because they have higher homogeneity, such as no significant differences in age and education among 18–30 year olds. This group is known to be active both in society and on the Internet and, thus, quite likely to know about functional beverages. Face-to-face interviews, each of which took 20–25 minutes, were done in the fall of 2017 to gather the data, and no intermediaries were involved. Responses were carefully checked for completeness, and students were informed that they could refuse to participate or refrain from answering any particular questions that they considered overly sensitive. Students were not paid for their participation, and replacements were found for those declining to participate. The data was gathered mainly on campus, either in the classroom or at other campus locations. In total, we received 261 responses. However, only 213 responses were considered for analysis after invalid responses were removed.

As Table 2 shows, the majority of the participants were female (74.65%). A large percentage were seniors (43.66%), and the most common purchase frequency was 1–2 times a week (38.50%).

### 2.5. Statistical Analysis

The theoretical framework was analyzed using SPSS (Statistical Package for Social Science) (IBM Corp.: New York, NY, USA) and AMOS (Analysis of Moment Structure) version 21 (IBM Corp.: New York, NY, USA). Two SEM study models were investigated in a study [51]. The two models, namely a measurement model and a structural model, were used to test for validity and reliability, and to test for model fit and hypothesis testing, respectively.

## 3. Results

### 3.1. Measurement Model: Reliability and Validity

Nunnally [51] noted that there is high internal consistency when Cronbach’s α is higher than 0.7, and the measure should be rejected if Cronbach’s α is lower than 0.35. Further, if the factor loading in each construct is higher than 0.5, the construct has construct validity [52]. For the following questionnaire items, Cronbach’s α did not reach the standard of 0.5. The questions were deleted: concerning the health value, Item 2 “I think of myself as a person who is interested in healthy food” and Item 5 "I’m not concerned about the health-related consequences of what I eat”; concerning self-efficacy, Item 25 "It is easy for me to buy functional beverages”, Item 26 “Drinking functional beverages is under my control”, and Item 27 “I believe that drinking functional beverages has a positive impact on my health." Since most of the questionnaire items had a Cronbach’s α higher than or close to 0.7, there was high reliability. Furthermore, the average variance extracted (AVE) and construct reliability (CR) also met the criterion. The details of reliability and convergent validity are outlined in Table 3. Means, standard deviations, and correlations among the constructs are presented in Table 4.

### 3.2. Structural Model: Goodness of Fit Statistics and Hypothesis Testing

A goodness of fit test conducted on the theoretical framework yielded the following results, which lie within the acceptable limits (x^2^ = 242.762, x^2^/df = 2.352, goodness of fit index (GFI) = 0.916, Tucker Lewis index (TLI) = 0.927, incremental fit index (IFI) = 0.954, root mean square error of approximation (RMSEA) = 0.046). All other fit indices were above the recommended criteria [53]. As a result, all indices provided evidence of an acceptable measurement model (Table 5).

### 3.3. Results of SEM 

**Figure 2:**
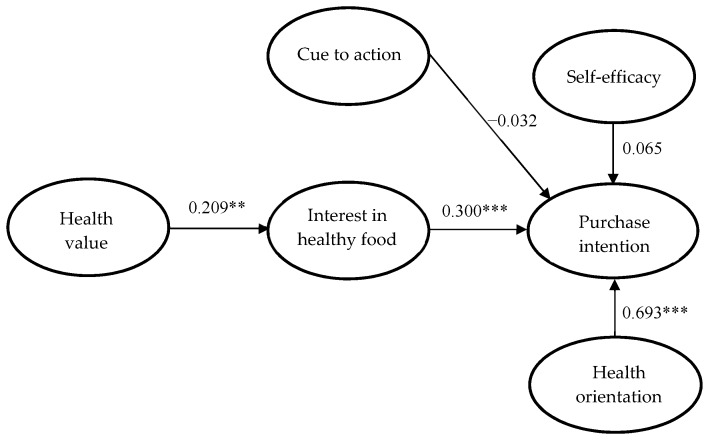
Results of SEM. Note.** *p* < 0.01; *** *p* < 0.001

The results of the participants’ purchase intentions concerning functional beverages are shown in Figure 2. Healthy value had a positive influence on interest in functional beverages (β = 0.209, *p* < 0.05), and interest in healthy food had a positive significant influence on purchase intention (β = 0.300, *p* < 0.001). Health orientation had a positive significant influence on purchase intention (β = 0.693, *p* < 0.001). However, neither cue to action (β = −0.032, *p* > 0.05) nor self-efficacy (β = 0.065, *p* > 0.05) had a positive influence on purchase intention. Based on these findings, H1, H2, and H5 were supported, but H3 and H4 were not.

## 4. Discussion

A summary of the verification of the hypotheses made in this study is shown in Table 6. The results of this study supported H1, given that the participants’ interest in healthy food was significantly affected by the health value. It appeared that the participants paid much attention to their health and were interested in functional beverages with the “little green label” (a green label attached to healthy products certifying specific health efficacy in Taiwan). This finding is in agreement with Olsen [30] results showing that there is a strong relationship between consumers’ health value and attitude in healthy products.

With regard to H2, interest in healthy food and purchase intention appeared to be highly related in the sense that as interest in functional beverages rose, so did purchase intention. This result clearly supports the notion that the more concern consumers show, the higher the possibility is that they will choose healthier products [42,43]. 

However, contrary to H3 and H4, the analysis showed that neither cue to action nor self-efficacy was a significant predictor of purchase intention. This might indicate that for consumers, extrinsic cues (e.g., advertisements, lectures) are not a critical factor in purchase intention for functional beverages. These findings are not in accord with the results of previous studies [7]. Strecher et al. [54] results were showing that strong relationships between self-efficacy and health behavior change and maintenance. Matthews, Doerr, and Dworatzek [55] results were showing that higher self-efficacy, and more cue to action toward healthier eating. Warziski et al. [56] showed that self-efficacy for controlling eating in a set of circumstances increased with weight loss and was correlated to the degree of weight loss. From the findings on cue to action and self-efficacy, we found that the participants had insufficient or quite different cognition about functional beverages. Analyze the reason and it may be related to nearly 75% of the female respondents in this study, they tended to consider drinks as unhealthy.

Lastly, health orientation had a positive influence on purchase intention (H5). This is in line with De Marchi et al.’s [28] results showing that health-related actions and food consumption decisions resulted from health orientation. The more consumers understand information about functional beverages, the higher their purchase intentions might be.

## 5. Conclusions

### 5.1. Conclusions

The results of this study show that the health value, interest in healthy food, and health orientation have a significant influence on consumers’ willingness to purchase health products. This means that the more consumers care about their health, the healthier life they tend to have, and the more robust products they choose. Thus, this study suggests that manufacturers can formulate a sound marketing strategy to motivate consumers’ health awareness and increase their interest in healthy food. For example, slogans or advertisements might ask, “Is the beverage you are drinking healthy?” or “Are you healthy?”. Beverages manufacturers might also feature information about health value on product packaging to arouse consumers’ interest in healthy food. Once these strategies succeed in motivating consumers to pay attention to their health, the consumers will be more interested in health products and have greater purchase intention.

### 5.2. Management Implications

From the findings on cue to action and self-efficacy, we found that the participants had insufficient or quite different cognition about functional beverages. They tended to consider drinks as unhealthy. Yet foods or beverages that are certified as having a health function may help improve their health. To eliminate the “unhealthy” perception of functional beverages, this study suggests that the government should promote the benefits of drinking water or the significance and importance of health labels such as the "little green label." It is necessary to establish common knowledge about beverages and change people’s perceptions by holding food safety education briefings. Another suggestion is to promote the meaning of relevant certifications through public media. Once consumers have accurate, detailed knowledge about how to identify related healthy labels, their purchase intention may increase.

In recent years, consumers have increasingly been paying attention to healthy diets, and their health values, interest in health food, and health orientation affect their willingness to purchase healthy products. Consumers who care more about health tend to have healthier lives and choose more robust products. Furthermore, related industries can keep upgrading the health functions of their products, for example, to regulate blood pressure, blood lipids, and blood sugar. Moreover, associated industries may apply healthy food labels or certification to highlight how healthy their products are and explain the product specifications so that consumers may choose functional beverages according to their own needs and increase their purchase intention.

### 5.3. Research Limitations and Further Research

This study only focused on college students in central Taiwan, and the sample was not large enough to represent all university students’ cognition and behavior on functional beverages. Additionally, since most of the participants were female, the results may not accurately represent how men think about functional beverages. Therefore, more extensive research (e.g., with an expanded investigation area and more excellent age range) is needed in the future. Other statistical variables (e.g., monthly income) or other variables (e.g., personality, lifestyle) may be added as well, to explore whether they affect a consumer’s purchase intention for functional beverages, making the research framework more complete.

## Figures and Tables

**Table 1:** Constructs/variables and corresponding measuring statements included in the questionnaire.

Construct/Variable	Number of Statements	Measuring Items	Sources of Adoption
Health value	3	I often think about my health. I think of myself as a person who is interested in healthful food. Good health is important to me.	Tudoran et al. [31]
Interest in healthy food	15	I think buying functional beverages is a good idea. I think buying functional beverages is very important. I think buying functional beverages is good. I think buying functional beverages is wise. I think buying functional beverages can regulate blood lipids. I think buying functional beverages can regulate blood sugar. I think buying functional beverages can improve osteoporosis. I think buying functional beverages can improve the immune system. I think buying functional beverages can improve gastrointestinal function. I think buying functional beverages can protect the liver. I think buying functional beverages can regulate blood pressure. I think buying functional beverages has a body fat lowering effect. I think buying functional beverages can help improve allergies. It is essential to have functional beverages every day. I believe that buying functional beverages can keep my body in shape.	Tudoran et al. [31]
Cue to action	3	I read about functional beverages and understand that drinking functional beverages can improve your body function. There are lectures on functional beverages at my university. There are advertisements or articles posted on functional beverages at my university.	Vassallo et al. [20]
Self-efficacy	4	I have set clear goals to improve my health. I have achieved my goals to improve my health. I am trying to improve my health. I think I have control and understand my health.	Şimşekoğlu and Lajunen [47], Lee, Hwang, Hawkins, and Pingree [48].
Health orientation	3	I’m interested in information on functional beverages. I often pay attention to functional beverages. I often read information about functional beverages.	Ng, Kankanhalli, and Xu [49]
Purchase intention	5	If functional beverages are available, I will try to buy one. If I choose again, I will still buy functional beverages. I try to buy functional beverages because they are the best choice. I think I am a loyal customer of functional beverages. I’m happy to buy functional beverages.	Lee, Hsu, Han, and Kim [50], Yazdanpanah et al. [7].

**Table 2:** Sample characteristics.

*N* = 213	Item	*N*	Percentage	Variable	Item	*N*	Percentage
Gender	Male	54	25.35%	The frequency of buying functional beverages	Never	24	11.27%
	Female	159	74.65%		1–2 times a week	82	38.50%
Level	Freshman	33	15.49%		3–4 times a week	18	8.45%
	Sophomore	32	15.02%		More than 5 times a week	5	2.35%
	Junior	31	14.55%		1–2 times a month	55	25.82%
	Senior	93	43.66%		1–2 times semi-yearly	22	10.33%
	Graduate student	22	10.33%		1–2 times a year	7	3.29%
	Other (Department of Medicine)	2	0.94%				

**Table 3:** Results of the factor loading, reliability, and validity.

Constructs	Items	Factor Loading	Cronbach’s α	CR	AVE
Health value (HA)	HA1	0.578	0.825	0.842	0.648
	HA2	0.921			
	HA3	0.873			
Interest in healthy food (IHF)	IHF1	0.617	0.941	0.923	0.522
	IHF2	0.605			
	IHF3	0.608			
	IHF4	0.581			
	IHF5	0.825			
	IHF6	0.850			
	IHF7	0.804			
	IHF8	0.805			
	IHF9	0.730			
	IHF10	0.813			
	IHF11	0.868			
	IHF12	0.730			
	IHF13	0.727			
	IHF14	0.595			
	IHF15	0.567			
Cue to action (CA)	CA1	0.549	0.802	0.823	0.619
	CA2	0.811			
	CA3	0.948			
Self-efficacy (SE)	SE1	0.874	0.860	0.859	0.606
	SE2	0.771			
	SE3	0.724			
	SE4	0.735			
Health orientation (HO)	HO1	0.805	0.890	0.893	0.737
	HO2	0.935			
	HO3	0.830			
Purchase intention (PI)	PI1	0.753	0.924	0.909	0.668
	PI2	0.884			
	PI3	0.872			
	PI4	0.781			
	PI5	0.787			

Note. CR = Composite reliability, AVE = Average variance extracted.

**Table 4:** Means, standard deviations, and correlations of constructs.

Construct	Mean	S.D.	1	2	3	4	5	6
1. Health value (HA)	5.96	0.92	1.00					
2. Interest in healthy food (IHF)	5.12	1.30	0.22	1.00				
3. Cue to action (CA)	4.39	1.32	0.27	0.35	1.00			
4. Self-efficacy (SE)	4.90	0.96	0.38	0.32	0.30	1.00		
5. Health orientation (HO)	5.34	1.08	0.47	0.45	0.31	0.40	1.00	
6. Purchase intention (PI)	5.72	1.16	0.50	0.44	0.39	0.33	0.48	1.00

Note. S.D. = Standard Deviation.

**Table 5:** Summary of goodness-of-fit indices for the structural models.

Model	*x* ^2^	*x*^2^/df	GFI	TLI	RMSEA	IFI
Structural model	242.762	2.352	0.916	0.927	0.046	0.954
Recommended value	N/A	≦3.00	≥0.90	≥0.90	<0.08	≥0.90

Note. goodness of fit index (GFI) = 0.916; Tucker Lewis index (TLI) = 0.927; incremental fit index (IFI) = 0.954; root mean square error of approximation (RMSEA) = 0.046.

**Table 6:** Summary of hypothesis verification.

Hypothesis	Content	Verification
H1	Health value has a positive influence on interest in functional beverages.	Supported
H2	Interest in healthy food has a positive influence on purchase intention.	Supported
H3	Cue to action has a positive influence on purchase intention.	Rejected
H4	Self-efficacy has a positive influence on purchase intention.	Rejected
H5	Health orientation has a positive influence on purchase intention.	Supported
